# Supplementary material for: Ultrarobust, tough and highly stretchable self-healing materials based on cartilage-inspired noncovalent assembly nanostructure
Source: Nat Commun. 2021 Feb 26;12:1291. doi: 10.1038/s41467-021-21577-7 (PMC7910491; doi:10.1038/s41467-021-21577-7)
Supplement: Supplementary file 1 — Supplementary Information [file 41467_2021_21577_MOESM1_ESM.pdf]

Supplementary Information

Ultrarobust, tough and highly stretchable self-healing materials based on cartilage-inspired noncovalent assembly nanostructure

Yuyan Wang[1], Xin Huang[1] & Xinxing Zhang[1]*

[1] State Key Laboratory of Polymer Materials Engineering, Polymer Research Institute, Sichuan University, Chengdu 610065, China
Correspondence and requests for materials should be addressed to X.Z. (email: xxzwwh@scu.edu.cn)

Supplementary Figures:

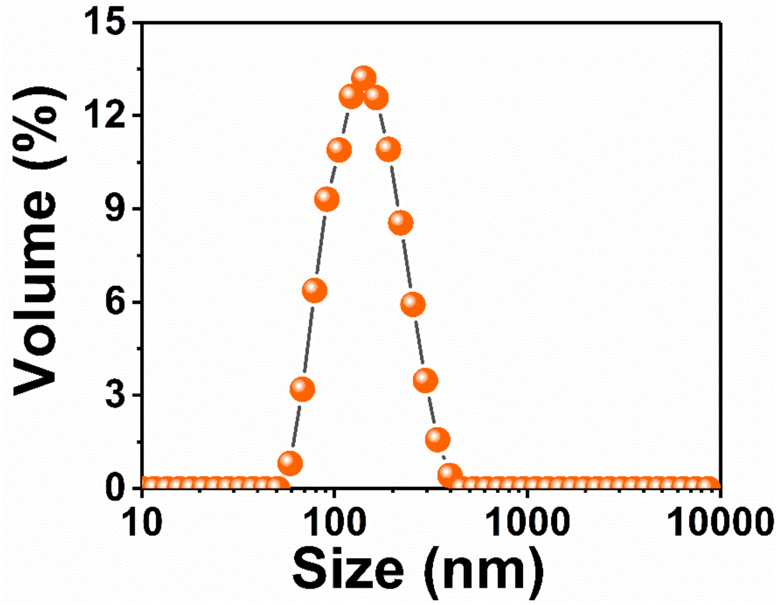

Supplementary Figure 1. Particle size distribution of WS_2 nanosheets.

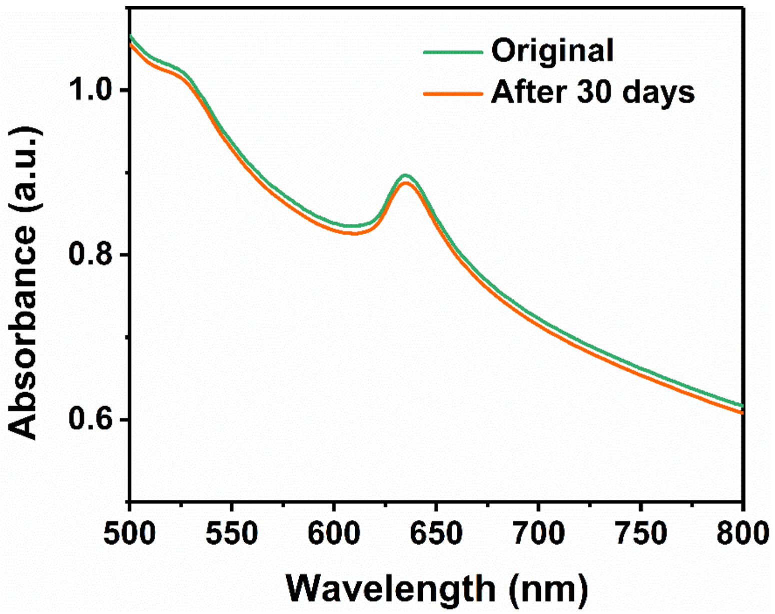

Supplementary Figure 2. UV–vis absorption spectra of the exfoliated WS_2 nanosheets in TA solution after incubated 0 day and 30 days.

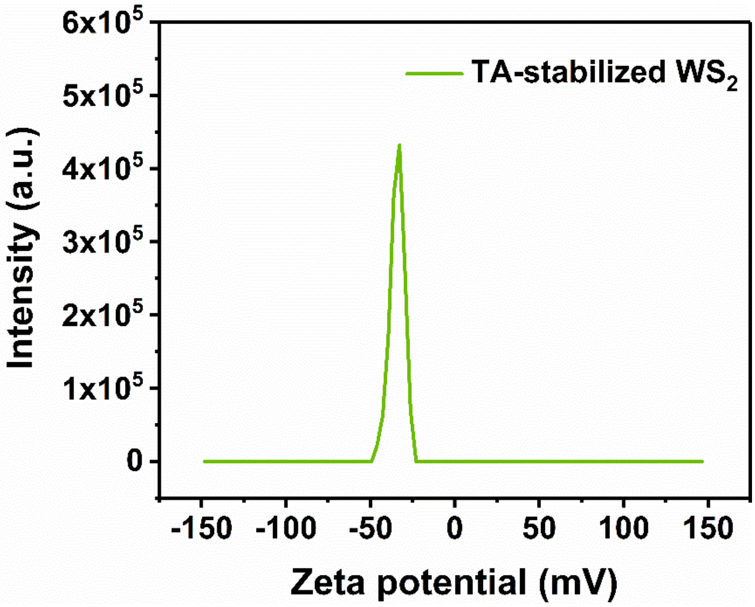

Supplementary Figure 3. Zeta potentials of WS_2 nanosheets in TA solution.

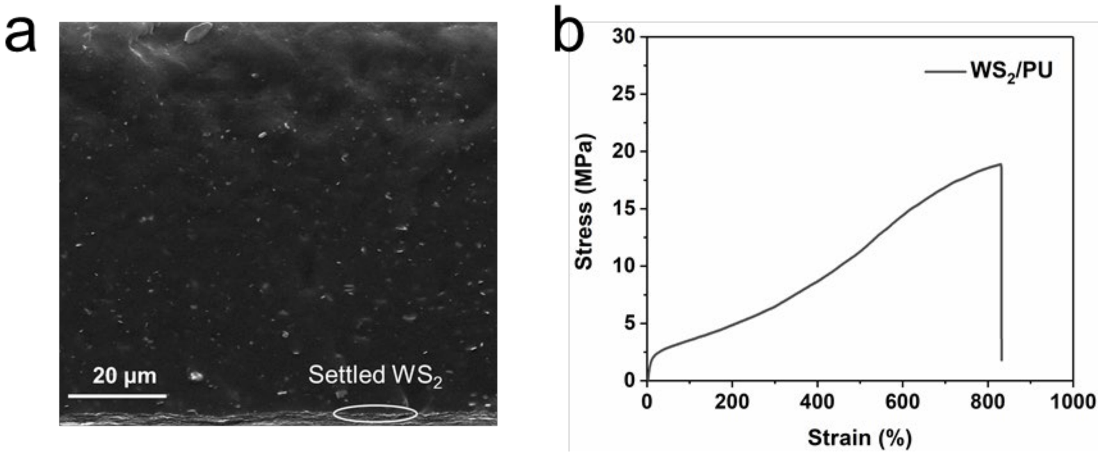

Supplementary Figure 4. a A SEM image of the cross section of 16%WS_2/PU composite. b Stress-strain curves of 16wt%WS_2/PU.

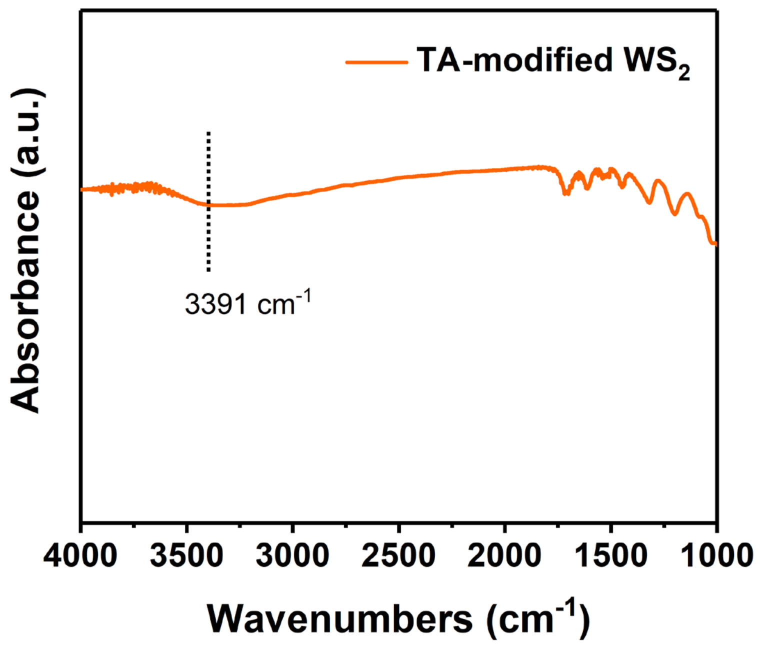

Supplementary Figure 5. FTIR spectra of WS_2 nanosheets dispersion after one week of dialysis.

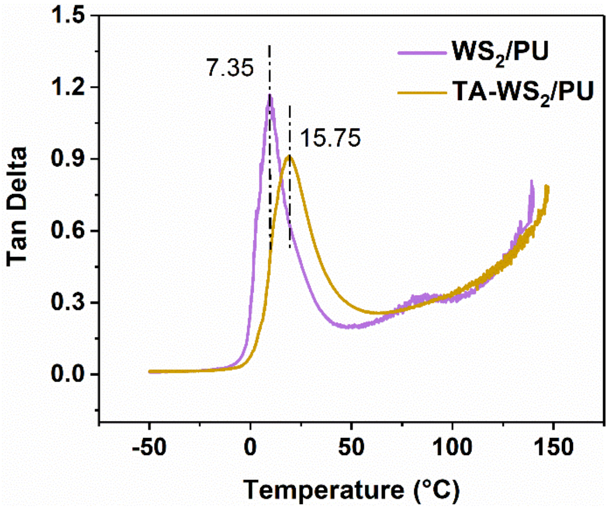

Supplementary Figure 6. Dependence of tanδ on temperature for WS_2/PU and TA-WS_2/PU composites.

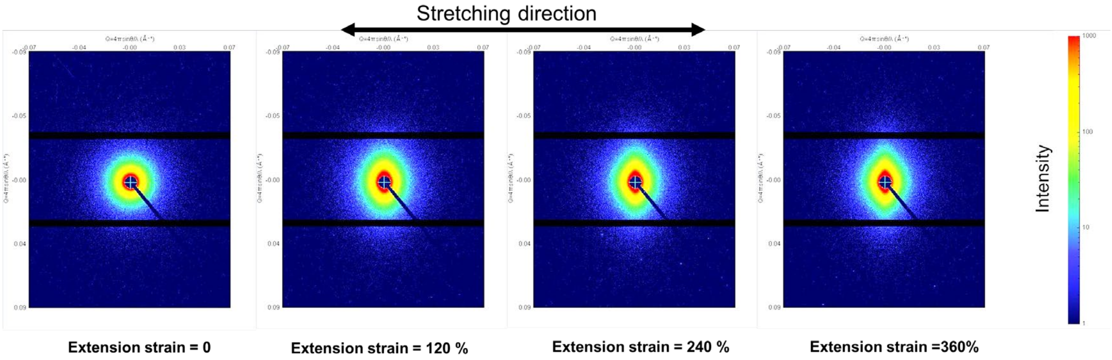

Supplementary Figure 7. 2D SAXS images of the TA-WS2/PU with different extension strain during uniaxial stretching process.

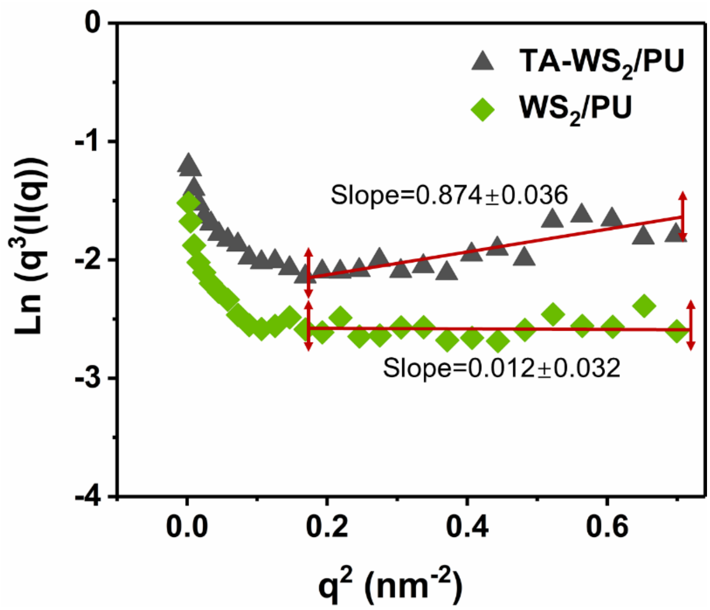

Supplementary Figure 8. SAXS plots of ln(I(q)) versus ln(q); origin data were divided into two parts according to the slope, and those slope values are represented in the graph.

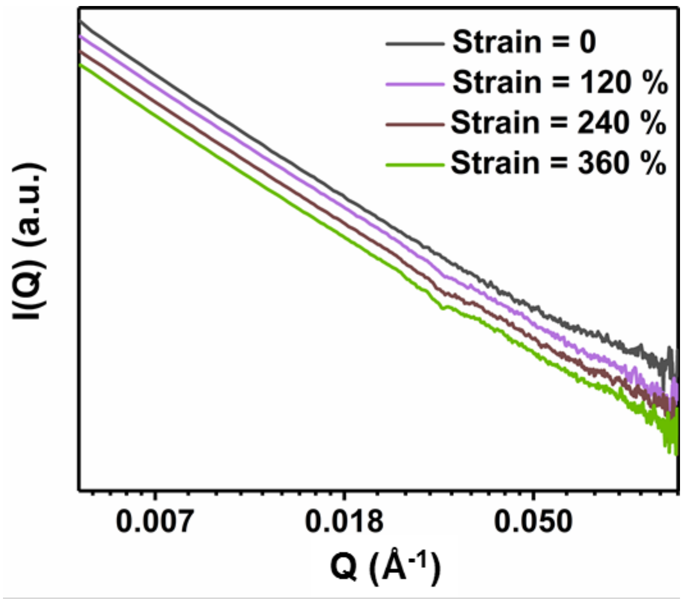

Supplementary Figure 9. 1D scattering profiles of WS_2/PU integrated from 2D SAXS patterns under different strains.

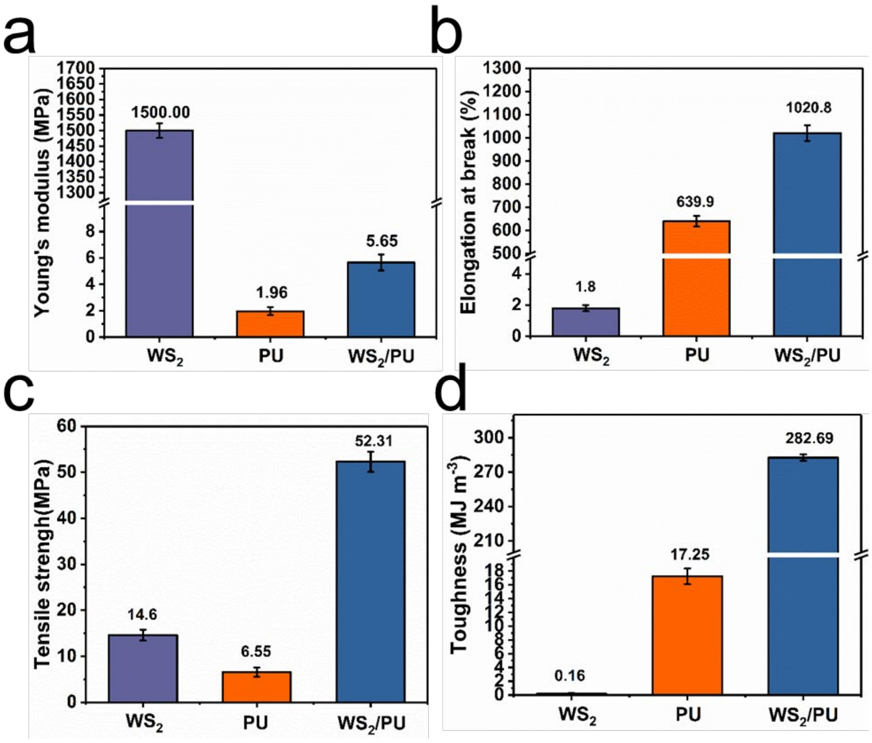

Supplementary Figure 10. Comparison of (a) Young's modulus, (b) elongation at break, (c) ultimate tensile strength, and (d) toughness of the pure WS2, PU, and 16%TA-WS2/PU nanocomposite.

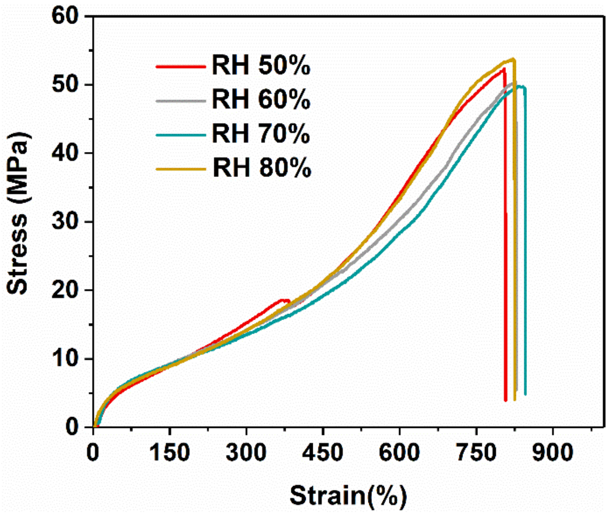

Supplementary Figure 11. Stress-strain curves of 16%TA-WS_2/PU under different humidity (RH 50%, 60%, 70%, 80%).

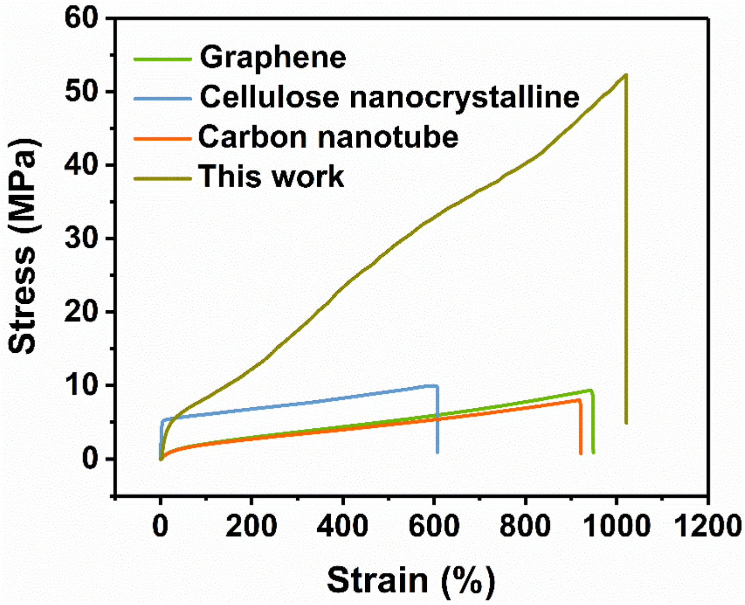

Supplementary Figure 12. Stress-strain curves of PU composites filled with different rigid fillers.

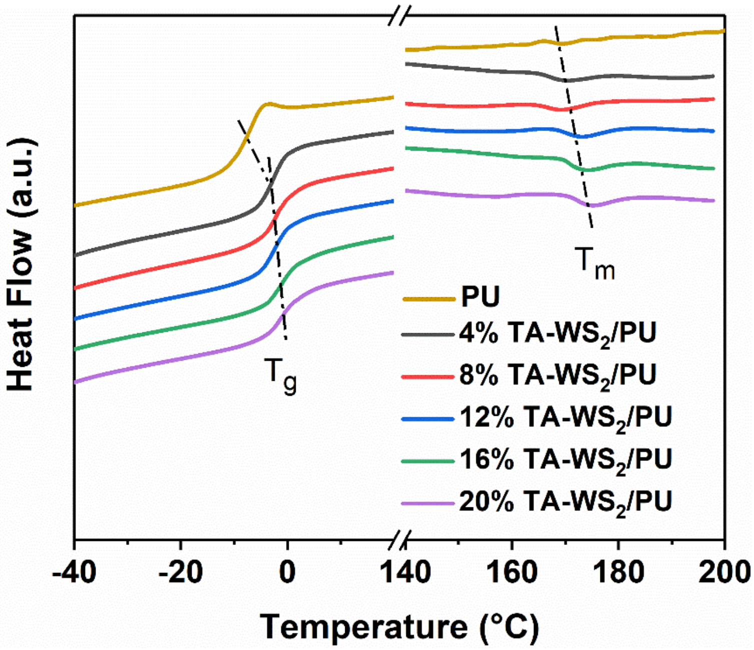

Supplementary Figure 13. DSC study of PU with different contents of TA-WS_2.

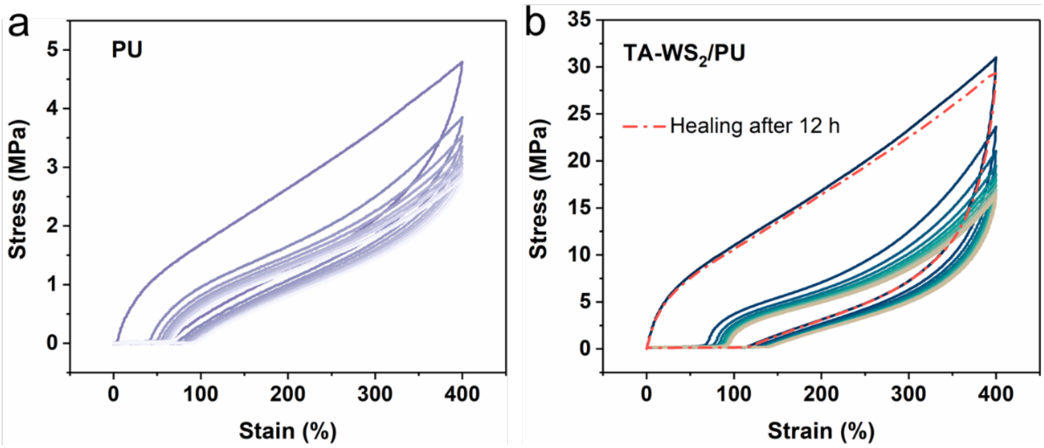

Supplementary Figure 14. Cyclic loading and recovery of PU (a) and hybrid elastomer (16% TA-WS_2) (b). The samples were conducted cyclic loading for ten times (400% strain) at a tensile rate of 100 mm min^{-1}, and then the damaged hybrid elastomer was healed 12 h at room temperature and stretched again (cycle 11).

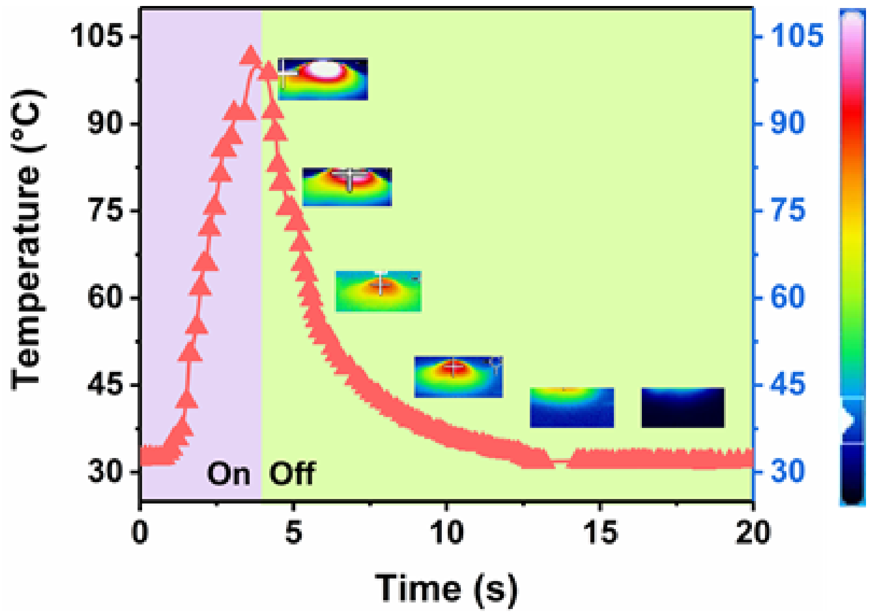

Supplementary Figure 15. NIR light-induced temperature rise of TA-WS_2/PU film, the images inserted in the upper right corner are temperature distribution IR camera images of a sample in the heat transfer process.

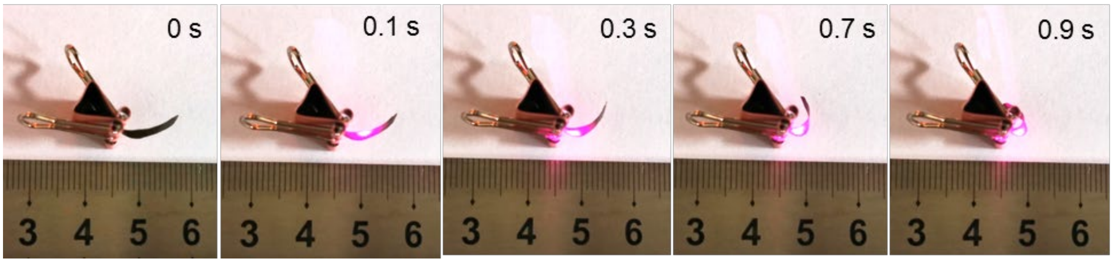

Supplementary Figure 16. Photographs of a film (15 mm × 2 mm × 0.15 mm) bending with NIR light (808 nm) irradiation.

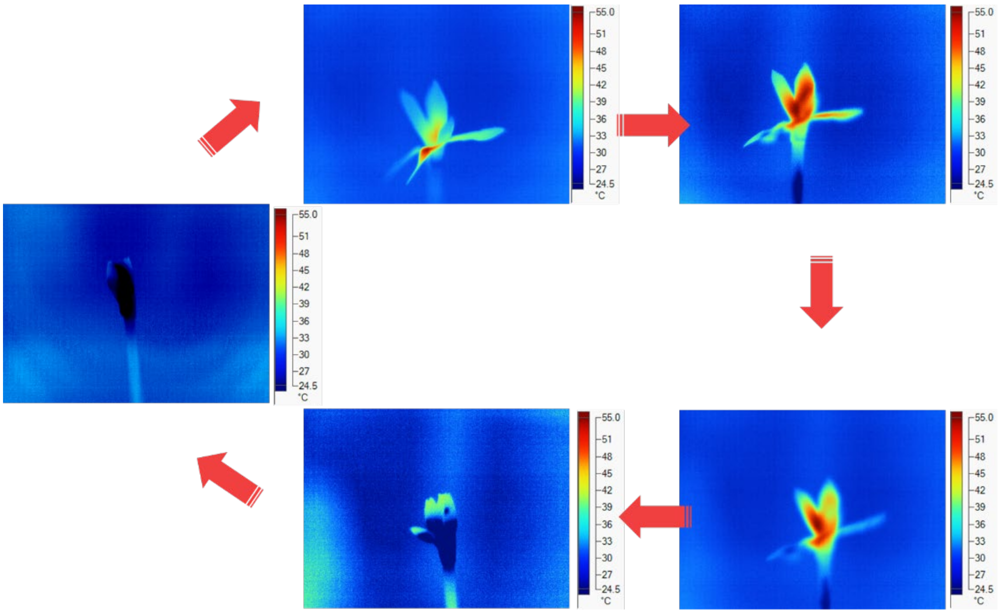

Supplementary Figure 17. Temperature distribution IR camera images of the "flower" in the heat transfer process.

Supplementary Table:

Table 1. Comparison of UTS, elongation at break, toughness, and recovery tests of various self-healing polymers [a].

Ref.	Self-healing motif	Ultimate tensile Strength [MPa]	Elongation at break [%]	Toughness [MJ m^{-3}]	Self-healing efficiency [%]	Functional healing	Self-healing conditions
1	Disulfides	6.8	923	26.9	76	yes	RT. 2 h
2	Metal–ligand	1.7	1700	15.3	78	\	RT. 48 h
3	Metal–ligand	1.1	310	1.35	76	\	RT. 48 h
4	Ag–S bonds	0.6	1200	3.6	93	yes	NIR <1 min
5	Olefin	17.7	1270	68.2	59	\	RT. 120 h
6	Hydrogen bonding	25	1.85	0.2	95	\	green laser at 80 °C 6 min
7	Disulfides	10	550	28.9	96	\	Under sunlight in July. 6 h
7	Disulfides	10	550	28.9	43	\	RT. 6 h

[8]	Metal–ligand	10	607	46.1	92	yes	RT. 15 s
This work	Hydrogen bonding	52.3	1021	282.7	80.6	yes	RT. 12 h
		35.8	1127	225.5	105.1	yes	RT. 12 h

[a] RT stands for room temperature, self-healing efficiency = $\sigma_{healing}/\sigma_{original}$.

Supplementary Note:

Note 1. The robot first stretches into the plane due to the asymmetrically photothermal expansion. Force analysis reveals that the horizontal traction force of the rear-leg can exceed its maximum static friction resistance to move forward (Fig. 4f, position I). When moving to the position III, the stretched robot begins to bend to its original shape due to the NIR ligh off. According to the force analysis, the horizontal traction force of the front-leg (which is larger than the horizontal force of the rear-leg) will exceed its friction resistance, so the front leg will move forward until a new force balance is reached. As a result, this robot can crawl under periodic NIR light on and off.

Supplementary References

1. Kim, S. M. *et al.* Superior Toughness and Fast Self-Healing at Room Temperature Engineered by Transparent Elastomers. *Adv. Mater.* **30**, 1705145 (2018).
2. Kang, J. *et al.* Tough and Water-Insensitive Self-Healing Elastomer for Robust Electronic Skin. *Adv. Mater.* **30**, 1706846 (2018).
3. Rao, Y. L. *et al.* Stretchable self-healing polymeric dielectrics cross-linked through metal-ligand coordination. *J. Am. Chem. Soc.* **138**, 6020–6027 (2016).
4. Song, P., Qin, H., Gao, H. L., Cong, H. P. & Yu, S. H. Self-healing and superstretchable conductors from hierarchical nanowire assemblies. *Nat. Commun.* **9**, 1–9 (2018).
5. Wang, H. *et al.* Synthesis of Self-Healing Polymers by Scandium-Catalyzed Copolymerization of Ethylene and Anisylpropylenes. *J. Am. Chem. Soc.* **141**, 3249−3257 (2019).
6. Si, Q. *et al.* Controllable and Stable Deformation of a Self-Healing Photo-Responsive Supramolecular Assembly for an Optically Actuated Manipulator Arm. *ACS Appl. Mater. Interfaces* **10**, 29909–29917 (2018).
7. Xu, W. M., Rong, M. Z. & Zhang, M. Q. Sunlight driven self-healing, reshaping and recycling of a robust, transparent and yellowing-resistant polymer. *J. Mater. Chem. A* **4**, 10683–10690 (2016).
8. Wang, Y. *et al.* Hierarchically Structured Self-Healing Actuators with Superfast Light- and Magnetic-Response. *Adv. Funct. Mater.* **29**, 1906198 (2019).
