# Supplementary material for: Ultrarobust, tough and highly stretchable self-healing materials based on cartilage-inspired noncovalent assembly nanostructure
Source: Nat Commun. 2021 Feb 26;12:1291. doi: 10.1038/s41467-021-21577-7 (PMC7910491; doi:10.1038/s41467-021-21577-7)
Supplement: Supplementary file 3 — Description of Additional Supplementary Files [file 41467_2021_21577_MOESM3_ESM.pdf]

Description of Additional Supplementary Files

File Name: Supplementary Movie 1
Description: a “flower” blooming and closing

File Name: Supplementary Movie 2
Description: a “crawling robot” moving forward
